# Health Sciences students’ experience of COVID-19 case management and contact tracing in Cape Town, South Africa

**DOI:** 10.1186/s12909-023-04205-4

**Published:** 2023-04-12

**Authors:** Virginia Zweigenthal, Gonda Perez, Karen Wolmarans, Lorna Olckers

**Affiliations:** 1 School of Public Health, Faculty of Health Sciences, University of Cape Town, Town, South Africa; 2 Department of Medicine, Faculty of Health Sciences, University of Cape Town, Town, South Africa

**Keywords:** COVID-19, Volunteers, Case and contact tracing, Health sciences education, Cape Town, South Africa

## Abstract

**Background:**

COVID-19 has challenged health and higher education systems globally. Managing the epidemic in Cape Town, South Africa (SA), required partnerships with universities and setting up of de novo systems for mass case and contact tracing (C&CT). Health sciences, predominantly medical students, as well as social work and psychology students formed the core of this telephone-based work over the 18 months when SARS-CoV-2 caused severe disease.

**Methods:**

This qualitative study aimed to elicit students’ motivations for becoming involved in C&CT, their experiences, and recommendations for C&CT and curricula. After Cape Town’s first COVID-19 wave, six on-line focus groups comprising 23 students were conducted, and a further four were conducted with 13 students after the second wave. As the researchers were predominantly educators previously involved in undergraduate health sciences education, the study’s purpose was to reflect on students’ experiences to make educational and health system recommendations.

**Results:**

Students were largely motivated to mitigate the impact of the epidemic on society and support people affected by COVID-19, as well as hone their professional skills. While these motivations were realised, students also needed to learn new skills – to autonomously work remotely, using novel communication strategies to engage those affected and use virtual groups to connect with colleagues. They managed responsibilities within the healthcare systems that did not always work smoothly, distressed cases who were financially insecure, difficult employers, and language barriers. They were prepared through training, and supported by virtual, yet effective teamwork and debriefing opportunities. Although the work was sometimes physically and emotionally exhausting, students found the work personally meaningful. They embraced public health’s role to protect population and individuals’ health.

**Conclusion:**

New teaching and learning practices adopted due to Covid-19 lockdowns enabled this digital C&CT project. It facilitated students to become confident, work autonomously and navigate challenges they will encounter as young professionals. The programme demonstrated that novel opportunities for rich student learning, such as in telehealth, can be embedded into public health and clinical functions of health services in contexts such as in SA, deepening partnerships between the health services and universities, to mutual benefit.

**Supplementary Information:**

The online version contains supplementary material available at 10.1186/s12909-023-04205-4.

## Background

South Africa (SA) was the African epicentre of the COVID-19 pandemic. In 2020, following World Health Organisation (WHO) guidelines, the country responded by preparing laboratory services, primary healthcare services, quarantine and isolation facilities, hospitals and field hospitals to manage the projected increased number of the sick [1], although operationalising this countrywide, was uneven. The Western Cape (WC) government, in a province that was heavily affected by COVID-19 from an early stage, additionally implemented a public health approach that included health messaging to prevent the spread of COVID-19, port-control, and a case investigation and contact tracing (C&CT) system to contain and slow community transmission through counselling cases to isolate and identifying contacts who needed support to quarantine. As was noted in many High Income Countries (HICs) of Europe [2], the United States of America (USA) [3], and in Lower to Middle Income countries (LMICs) such as Indonesia [4], there was insufficient personnel to perform this function for COVID-19. There was, however, a sense of urgency for this work in SA as the potential impact of COVID-19 was unknown. However, being a country already burdened by chronic and infectious diseases such as HIV and TB, the impact was likely to be great. The WC provincial Department of Health’s (PDoH) central electronic database of public sector patients was immediately repurposed to include positive COVID-19 laboratory results from both public and private pathology laboratories. This generated timeous lists of cases for follow-up [1].

The WC, the third largest provincial economy in SA, contributes 14% towards SA’s Gross Domestic Product, yet has the second highest per capita income [5]. Cape Town, the WC’s capital city with a 2020 population of 4.6 million [6], has the highest growth in GDP per capita, averaging 33% higher per capita compared to the national average in 2018 [7]. Nonetheless, 45.5% of residents live in poverty in this comparatively better resourced city. The WC health system is noted to be proactive, responsive and evidence-driven [8]. Consequently, managers and providers of public sector health services, delivered by the WC province and City, focused on the preparation of health services for the anticipated influx of sick COVID-19 patients. There were limited options to redeploy staff, contrary to what was done in European countries such as Serbia and Austria [2] or San Francisco, USA [9]. Consequently, volunteers were recruited to support the newly created C&CT system, and appeals for assistance were sent to the PDoH office-based employees, student societies [10] and health sciences faculties at universities with working relationships with the PDoH. These partnerships enabled university staff, retired academics, health care workers and student involvement in C&CT.

During the initial Level 5 lockdown, only essential workers were permitted to leave home. This resulted in retired and current academic staff and students volunteering to work in small teams, called ‘pods’, under the leadership of PDoH Public Health personnel. Initially this telephonic C&CT work was centralised in the WC provincial office, but from May 2020 the eight pods began to support the Cape Town district’s four devolved health administration substructures. Following the attrition of volunteers, who returned to work, from November 2020 onwards the pods combined into a “Metropod” during COVID-19’s Wave 2, eventually disbanding after the third COVID-19 wave in September 2021, when vaccination services were well established. The work was subsequently taken over by the provincial government’s Call Centre which worked with the Department of Health.

**Fig. 1:**
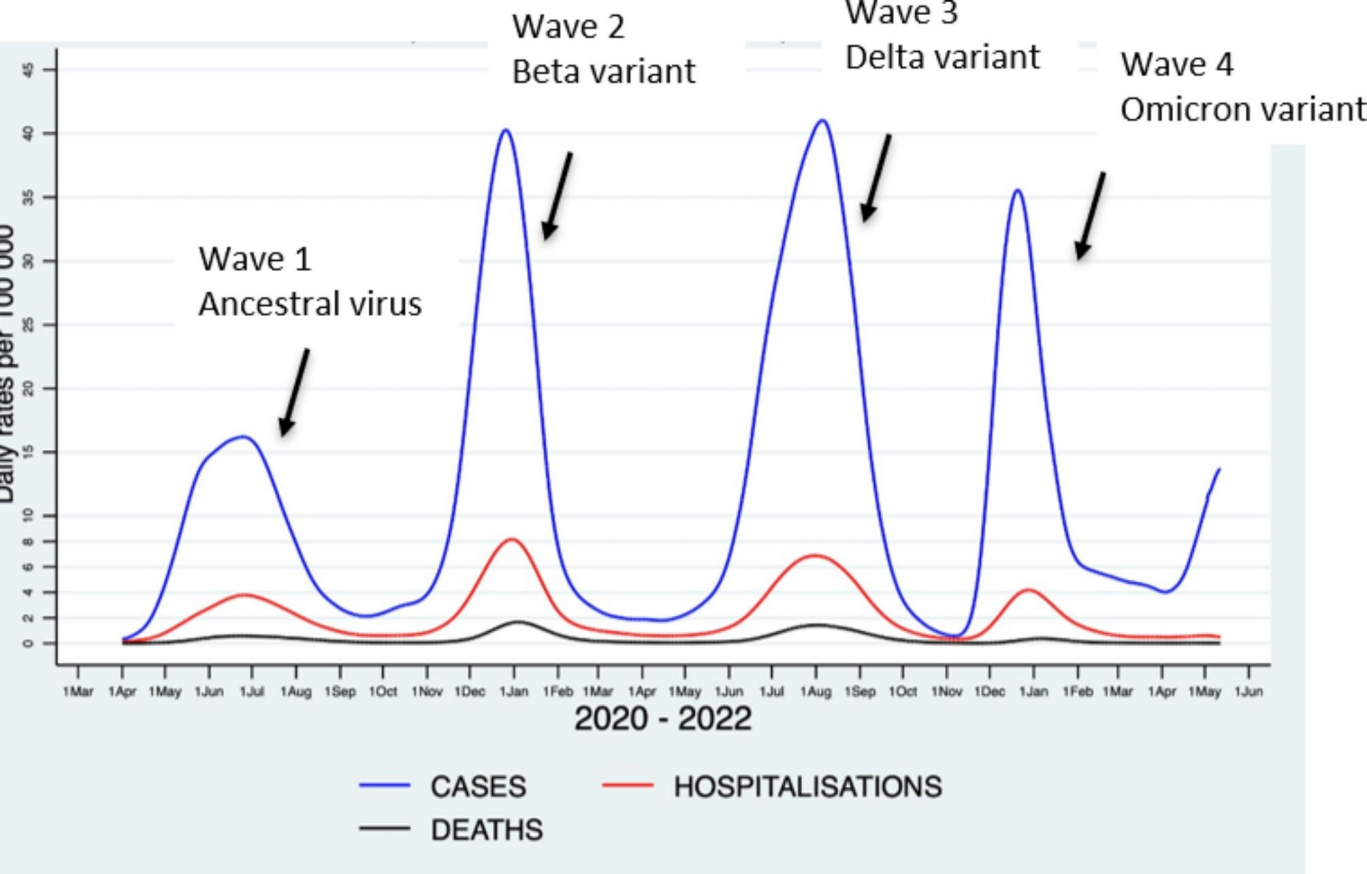
COVID-19 new cases, hospitalisations and deaths in the Western Cape (2020–2022)

As is seen in Fig. 1, SA’s COVID-19 waves were the result of the ancestral strain in Wave 1; the more transmissible and virulent variant, Beta, in Wave 2; and the yet more transmissible virulent Delta variant in Wave 3. Over the first two waves, vaccination was not yet available in SA, and these three variants resulted in severe disease.

The pods functioned seven days a week and volunteers worked long hours to timeously reach cases and their contacts, both to limit infection transmission as well as to mitigate the consequences of severe disease.

Student volunteers formed the backbone of the pods, and included under-graduate health sciences, social work, and psychology students, and post-graduate students in Public Health. Initially, students volunteered to work when universities closed, but after Wave 1, when university learning resumed on-line or in-person (for selected health sciences students), many discontinued their involvement. Medical students’ work then shifted from pure volunteerism (working for no benefit) to students electing to do the work in order to fulfil obligations of academic programmes. Over 200 students worked in C&CT during the first three waves of COVID-19 from March 2020 to September 2021, when the Metropod closed.

The work required volunteers to phone COVID-19 cases, ascertain their health status, comorbidities, details of their contacts, and offer support that included referrals to isolation (for cases) or quarantine (for contacts) facilities. Pod members provided letters for employers to enable cases and contacts to isolate or quarantine for the statutory mandated days; ordered food parcels for those who were food insecure; as well as offering advice to seek medical attention if cases experienced respiratory distress, and referrals to experienced counsellors where necessary. Public confidence, built in this first contact with the health system, meant that many volunteers were phoned at all hours of the day by anxious families of cases who had deteriorated and needed further care. Families had access to volunteers’ telephone numbers, as personal phones were used for this work.

### Training

As is detailed in Table 1, all volunteers, including students, were required to watch a video [11], to familiarise themselves with the suggested guide and sign a confidentiality pledge. They were then given access to a secure Google sheet that housed the line list of cases for each day’s work. Over time, additional orientation material, resources and support became available. By November 2020, improvements were implemented. Instead of emails, automated reporting forms were completed, fortnightly debriefing sessions were introduced, and all students were mentored by experienced volunteers. For students completing C&CT as a curricular elective, mentors gave students feedback on their written reflective assignments. In mid-2021, internet telephony options were made available to students to alleviate airtime costs.

**Table 1:** Orientation, training and support for students

Item		Content
Videos	Introduction	Epidemiology Clinical features Roleplay of interview
	Breaking bad news	Overview Frequently asked questions
Guide		Script for interviews – cases and contacts Resources on offer • Informatics to send to cases and contacts • Templates of letters for work and school Reporting (to substructure) via emails • spreadsheet of interview findings • request for home visit • request for isolation facility Request for food parcel to to PDoH, which contracted with NGO for delivery
**Improvements (December 2020)**		
Automation		• Reporting form • Food request • Isolation facility • Home visit
Guide		• Improved templates for work letters and school (cases and contacts)
Video		• Pod role, rosters, role of students, mentoring • Revised role play
Student support		• 1–1 orientation • 1–1 mentoring • Fortnightly debriefing sessions

### Motivation for the study

Historically, health science students’ education has been orientated to perfect competencies for the clinical encounter, with limited experience of working in cross-professional teams [12]. Health Sciences education in SA focusses on producing health professionals who are “fit for purpose” to tackle the country’s burden of disease [13], which largely means capable clinicians, with less emphasis on prevention and health promotion and public health related skills.

COVID-19 provided an opportunity for students to experience the importance of public health and functional interprofessional teamwork. This included confronting social determinants of health in the context of COVID-19; the meaning of primary and secondary prevention of disease – preventing viral transmission and the value of early detection of severe disease with prompt referral – while working in teams. At the same time students could exercise clinical skills in a novel way – over the phone. They used these skills in a context where telephonic consultations are increasingly becoming mainstream for health professionals as a communication modality [14]. This experience provided students with a unique learning opportunity which the researchers wanted to explore in detail. The researchers (VZ, LO, GP) were educators previously involved in undergraduate health sciences education, initiated and supported students in this COVID-19 programme. The study objectives were to explore students’ motivations for choosing to join the COVID-19 case management and contact tracing programme;; their experiences of working remotely; their recommendations for improving work processes and student learning with a view to making policy and educational recommendations, locally and globally.

## Methods

With the focus on student experiences, the study was framed within an interpretive theoretical paradigm that draws attention to how participants make sense of their experiences. This meant that qualitative methods were most appropriate[15, 16].

This qualitative research project involved focus group discussions, which were best suited to the study objectives, as focus groups can explore issues in depth, such as motivations, perceptions, and common experiences [17, 18]. A semi-structured flexible interview guide was designed that covered three domains – motivations, experiences, and recommendations [see Additional File 1]. Questions were carefully planned to open up the topic areas, and for more in-depth and deeper exploration of answers [19].

### Recruitment

Of the over 200 students who volunteered over the first two COVID-19 waves, the 157 with contact details were contacted by email and via WhatsApp, to invite them to participate in focus groups – 115 from Wave 1 and 42 from Wave 2. Those who expressed interest in participating were sent an information sheet, a consent form, and a link to an online form with time options to join a focus group discussion (FGD). All who signed up, were sent a link to join an online Zoom meeting via email. Consent forms, which included informed consent for recording, were emailed back to the researchers and checked prior to the focus group. One researcher conducted all six FGDs after the first COVID-19 wave, comprising 23 students in all, with 2–6 participants per group, with one of the other three researchers supporting in turn. After the second wave, another researcher conducted all four FGDs, comprising 2–4 students, with 13 students overall, again with a researcher in support. Focus groups lasted between 66 and 100 min each.

### Data management, analysis, and ethics

As this study was situated within an interpretive theoretical paradigm, thematic analysis was used. This involved an iterative process of identifying themes of interest and prevalence from the data, in order to compare and establish meaning [20–22]. Steps described by Braun and Clarke (2006) were followed, beginning with the researchers familiarising themselves with the data, and then moving onto generating initial codes, searching for themes, reviewing the themes, defining and naming final themes, and finally producing the report. Audio recordings of all focus groups, transcribed using Otter a.i., a transcription platform, were stored in a secure online drive, accessible only to the four researchers, who reviewed them, corrected errors, and anonymised participants. Researchers independently coded two transcripts inductively, for each domain of inquiry. Through discussion, the second step of establishing a preliminary list of codes was generated, and after all additional transcripts were coded, a final code list was generated. Codes were then reorganised into themes, and quotations that best spoke to these were identified. Each researcher drafted a theme, which was discussed as a research team. As required, the themes were reviewed through an iterative process to ultimately identify the final themes. The coherent research design – the identification of the research question, the data collection method selected, the data analysis process and reporting of rich findings, attest to the rigor of the research [23]. The quotes, in italics, are identified by the wave (W) the student worked in, the focus group number (G) and the student number (S), that is (W#G#S#). The research was approved by the Human Research Ethics Committee (HREC) at the University of Cape Town (HREC: 504/2020).

## Results

A total of 36 students participated in ten focus groups. As is shown in Table 2, most were medical students (75%). These students had volunteered during the first two waves of COVID-19 (April 2020 - April 2021) at a time when infection with SARS-CoV-2 resulted in severe outcomes and no vaccinations were available.

**Table 2:** Students’ educational background

Course/year	Total n (%)
Medicine	27 (75.0%)
Year 1–3	5 (13.9%)
Year 4–6	22 (61.1%)
Occupational therapy	2 (5.5%)
Year 2	1 (2.8%)
Year 4	1 (2.8%)
Public Health (post-graduate)	2 (5.5%)
Speech Therapy: year 3	1 (2.8%)
Social work: year 3	1 (2.8%)
Social science (undergraduate): year 3	1 (2.8%)
Unknown	2 (5.5%)
**Total**	**36 (100%)**

**Fig. 2:**
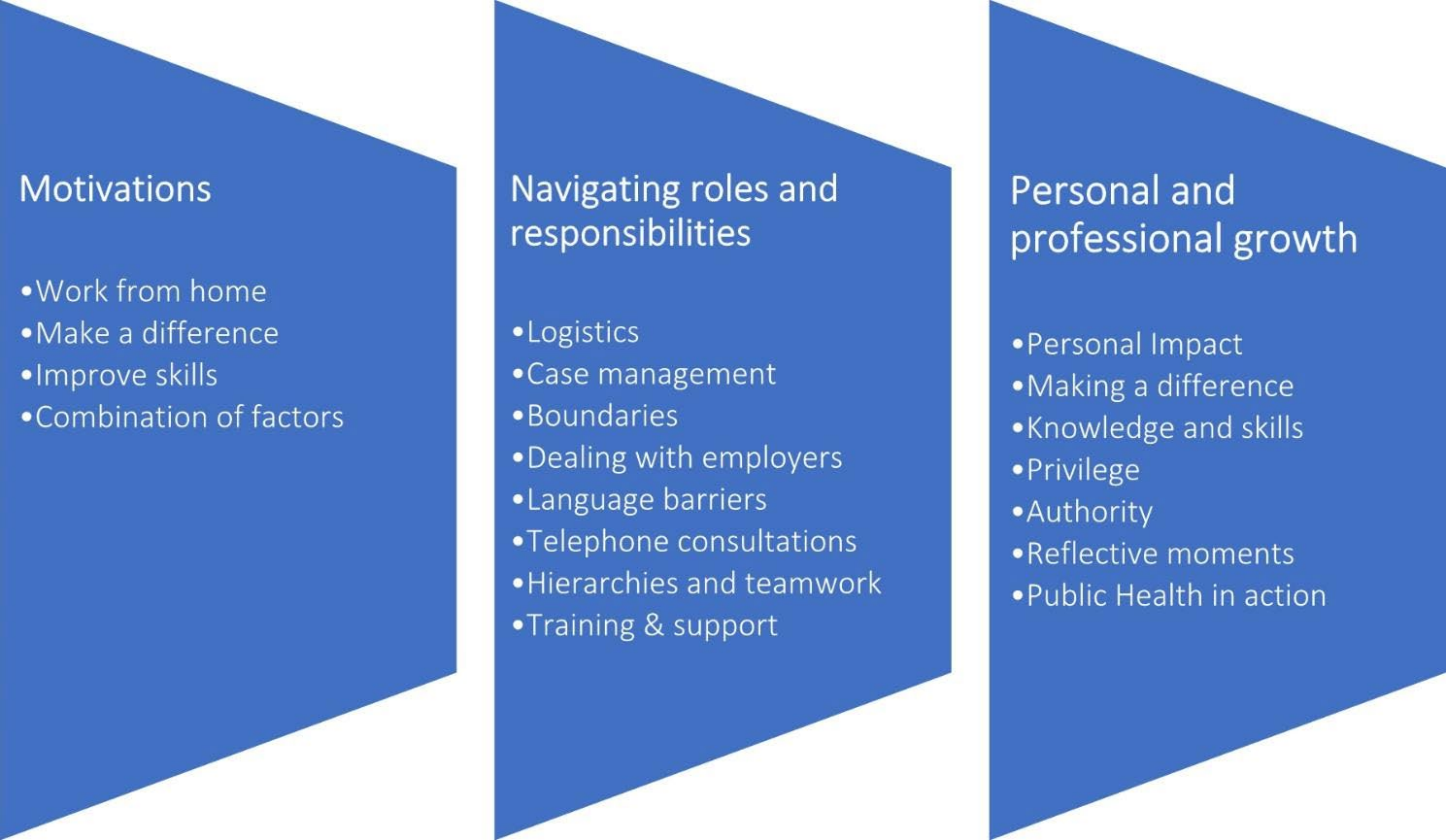
Outline of themes

As is shown in Fig. 2, three overarching themes with subthemes were generated, particularly related to experiences.

### Motivations

The first broad theme was students’ motivations for becoming involved in C&CT. These ranged from being able to work from home, to making a difference, and honing their skills.

#### Work from home

At the start of the pandemic, universities were shut, and students returned home, often far away from Cape Town. While few were afraid of contracting COVID-19 themselves, many lived with older family members with co-morbidities placing them at risk of severe disease. Being able to work telephonically, from home, meant there was minimal threat to loved ones.

*Contact tracing was the best means of assisting and keeping [family] safe as well, rather than stepping out and putting them at risk as well as myself*. (W2G2S1)

*I thought that it would be a great opportunity to help out from home*. (W2G2S2)

#### Make a difference

Many students expressed an intense desire to make a difference. After infection had become established in communities, they heard about the epidemic’s toll, the demands put on front-line health workers in hospitals and the increasing deaths, including those of family members. Students wanted to contribute to mitigating the impact of COVID-19 particularly in disadvantaged communities. They spoke of wanting to feel useful and to do something meaningful that would overcome their sense of social isolation, which was frustrating and disempowering.

*I kind of felt lost in a way,... sudden change to being at home, the option of going out wasn’t there. Being a health professional and having taken an oath each and every year to want to do the society justice in every way possible. I think for me, that was … my drive.* (W1G6S1)

*My dad is a frontline worker. And being home and seeing how hard he was working and hearing the stories first-hand of the great need, really motivated me … There’s this doctor inside of me that thinks “I want to help and play my part and contribute to the great need, in our society and community in whatever way I can”*. (W1G4S5)

Many students reflected on their intended profession and believed they had skills to assist people which drew them into collective efforts to address COVID-19. For health sciences students, their identity as professionals-in-training was important, and they wanted to put skills into practice, to make a difference.

*I wanted to make a difference and, you know, be a part of something, especially when we have medical training behind it.* (W1G3S3)

*I signed up. I simply joined because I was missing the interaction between a health professional and any sort of patient really. So, I think it was really my longing for the clinical field.* (W1G4S1)

#### Improve skills

COVID-19 was a new disease and the learning curve for everyone involved was significant. Students expressed excitement at the opportunity to learn and improve their skills.

*It was quite an amazing opportunity to take responsibility. So, I think in med school, … usually our hands are held ... So, it’s quite interesting to be one-on-one with patients and be able to speak and have the responsibility that I’m giving the correct advice*. (W1G4S4)

By September 2020 university work resumed, and most who then joined the pods were medical students in their fifth or sixth years of study who needed a placement to complete their compulsory electives of either two or four weeks. Some wanted to use the opportunity to intentionally improve their confidence in interviewing and communication skills. Others wanted to hone skills in efficient history-taking, in understanding and managing people’s complex lives, or obtain a better understanding of public health.

*it was also like a matter of just practicing … interviewing skills… I felt it was an opportunity for me to … validate for myself.* (W1G6S1)

*So, I just thought like, it would help me to learn how to work with deeper issues, how to work with real life issues*. (W1G4S4)

#### Combination of motivations

For many students who volunteered, it was not one factor but a combination of factors, that made volunteering for remote C&CT attractive.

*And then I must admit, when they said, we could use this toward our elective, that was the cherry on top of contributing to society and be able to do something with my peers and having it benefit me as well as doing a greater good, was sort of my overall motivation*. (W1G4S5)

### Navigating roles and responsibilities

In all groups, students reflected on the roles and responsibilities they had to navigate. They described a range of challenges, but these were clearly balanced by excitement and experiences of teamwork, training, and support within the pods.

#### Logistics

Certain logistics associated with carrying out the responsibilities of C&CT were described as particularly challenging. Most often, this was incorrect phone numbers for cases on the daily spreadsheet, which was not unique to the student experience. Trying to find correct numbers was time-consuming, frustrating, and generated additional work – calling laboratories or health services for alternative patient numbers. This resulted in delays in getting to cases or leaving cases uncontacted.

*I think one of the main challenges I encountered was just trying to get hold of some numbers that weren’t answering. And I know I phoned quite a few of the hospitals to try and chase up another number. And I found that some of the hospitals were not always very forthcoming with patient numbers… which is understandable, but at the same time, it did make the job a little bit trickier.* (W2G3S3)

Another logistical challenge was that students were not informed when food parcels had arrived as requested or whether cases had arrived at isolation facilities. They did not always have adequate information to convey, when anxious cases or families of cases contacted them for updates, leaving students frustrated.

*I arranged a home visit and a food parcel, but it didn’t come on the Friday. They were calling me the whole weekend. And I thought maybe it will come on the Monday. And then on the Monday I had to apply for an isolation facility, food parcel and a home visit to check the situation there. But I think they only got there on Wednesday…. On Tuesday, they called me that the patient passed away in their room in the house.* (W2G3S1)

A further logistical challenge was when updated policies had not been adequately disseminated to healthcare providers and the public more generally or had not been properly understood. These included changes to lengths of isolation and quarantine, and concepts such as false positive or negative tests. Students felt the fallout as they had to explain these changes to cases and contacts.

*There was a lot of misconception and a lot of confusion about what the actual policy was, for… the evidence of being a case… You had to try and argue what we’ve been taught, but … it was quite tricky.* (W1G5S1)

*And if you have a positive test, there’s no point of testing again, for the hopes of getting a negative test … That was quite frustrating for me having to explain to people the process of false negatives*. (W2G2S2)

#### Case management

Students found managing cases and their responses to bad news, challenging. When the C&CT teams were set up, the expectation had not been for students and other volunteers to have to break the news of a positive COVID-19 result, but very quickly it became evident that the laboratories and health services were not managing to inform cases quickly enough. This meant that this responsibility often fell to students who, in turn, had to deal with a wide range of responses from cases – from denial to anger and fear. There was anger about who may have transmitted the virus to them; anxiety about managing difficult living situations with the demands of isolation; and fear of death. Students had to be adept at managing these various responses.

*There was such an array of reactions to testing positive... you would call, and they would be totally freaked out... you would have to take more of a … step back and make sure they’re feeling okay. And then … tell things to them in a much more gentle way.* (W1G6S1)

*So, when I broke the news to another case, she actually started crying. And that was a bit hard for me because … it was on the phone. And there was like nothing that I could do to comfort her. And then she also started asking me if she was going to die from COVID. So that was a lesson in … practicing empathy, being honest, but also like helping instilling hope in the patient at the same time.* (W1G5S2)

In a few instances, cases were afraid of the stigma of having contracted COVID-19 and did not want their friends or community members to know.

*The stigma around being infected with COVID was common.... And people were very afraid about the delivery of food packages and picking them up for isolation facilities; about whether it’s going to say “COVID-19” and the neighbours knowing.* (W1G1S3)

Students also found that conversations were difficult when individuals they contacted were very ill or in hospital. While they were trying to gather information, they had to be sensitive to the capacity of cases to engage. In some instances, cases had already died when students called, or died within days of their call. This was very difficult and often distressing.

*I called the case and, you know, and she said to me, no, I’m in hospital. And we had to ask, how are you? When were you admitted and all that stuff. And she was very open to having the conversation with me, you know, very cheerful lady... Around five in the evening... I’m texting to ask... “is everything okay?”. “She passed away”. And then you start asking yourself, Where did you go wrong? I ...call[ed] the ambulance centre... trying to get them a quicker ambulance... You know, these people are very vulnerable. So they’re bound to be a bit attached to you. And they look to you for assistance. They look to you for help... Then I just stopped … I don’t know. I think I managed.* (W1G2S1)

*I do think that even the documents can’t really prepare you for calling someone whose family member is deceased or for them calling you to tell you that a case you were following has passed on.* (W2G2S2)

#### Boundaries

Creating boundaries was also difficult for students as they were aware that they were often the one point of contact for cases who felt isolated and overwhelmed. Some found that they were pulled into ongoing contact with cases and their families. They became a link into the health service and a source of information particularly when family members were unable to reach loved ones. Cases were meant to be followed up by service-based case managers, but as numbers soared, ongoing contact with cases became increasingly difficult. This left students in a difficult position, as they were meant to be the initial contact service but took on more of the ongoing contact responsibilities.

*You … become the only point of contact for some people in terms of where they’re getting the information and the advice that they’re getting. Certain cases end up calling you quite often, just to … know what the next step is, especially when it’s for a family member.* (W2G2S2)

*People contacting me after I’d done...the main interview… because they didn’t get communication from the case manager ... they often resorted to contacting me back to sort out issues like work letters, and sick letters... I just kept having to give them reassurance, I just tried to re-explain the whole concept of the case manager and the fact that I was just the initial person contacting them, and that their case would be dealt with.* (W1G2S1)

Their own age and that of the cases they were calling was another issue. Some students seemed to find their own age concerning as they saw it as a measure of experience. Many felt relieved that cases did not know they were dealing with a young student. In contrast, students typically avoided calling elderly cases, being fearful of managing someone who may be very ill or may have died.

*I didn’t disclose my age ever. So they think … when you say you are from the Department of Health, they … assume that you are a professional.* (W1G2S3)

*If there was someone who’s 90 years old, I would not take that person, … I didn’t want to take that person, because I feared that perhaps they had passed away, or were… near approaching … the end of life. And I didn’t want to involve myself in that matter.* (W1G3S2)

One student described how having COVID-19 him/herself made a difference in terms of the ability to genuinely empathise with cases.

*Three days into contact tracing I got COVID. I was really compassionate, and I had empathy as I was literally in their shoes. (*W2G1S3)

#### Dealing with employers

As most of the cases contacted by the students were employed, students needed to communicate with their employers. They described this as challenging because of cases’ employment circumstances – employers who were not properly informed about COVID-19; those who did not appear to trust their employees; or, who showed disrespect towards employees.

*Oftentimes people’s employers would not be very understanding. And particularly if people were informally employed or employed as domestic workers… Often employers would be unreasonable in responding to their diagnosis. One case that stands out for me was a super-spreader case where the person had phoned, I think, almost 100 of her friends and contacts that she’d seen in the preceding week, to tell them to shut down … but hadn’t told her domestic worker that she had COVID, which was absolutely inexcusable. Completely shocking behaviour.* (W1G5S3)

#### Language barriers

The home language of many cases, living in affected working-class and vulnerable communities, was Afrikaans or Xhosa. Students expressed concern and frustration at not being proficient in languages other than their home language/s and the implications of this for the cases they called. They described how creativity was needed to bridge language divides. These included cases asking other family members to take the call and translate for them, or students moving between languages as best they could. Those, with local conversational language skills, found that they had to be careful to explain medical terminology in lay terms, so that cases were clear about what was being said. These language-related challenges had implications for time-management as this was often not straight-forward.

*I think my biggest challenge was the language barrier. I only have really basic isiXhosa from our training at varsity. And I think [that] in-person, perhaps it’s a little bit easier to communicate in a second or third language. Because you have body language* (W1G5S3)

*It’s one thing, being able to speak the language, conversationally, but it’s another thing having to explain concepts in that language, and especially like medical, scientific things. So … that was what was daunting for me… Once I actually got started and actually spoke to some cases, it … got easier... The cases themselves really appreciated … having someone who… speaks your home language... you’re still able … to find that common ground… I think that was really helpful and somewhat made them more receptive as well, to everything that we were sharing.* (W1G3S1)

#### Telephone consultations

Challenges with communicating with cases over the phone extended beyond the use of spoken language. As they did not see cases in person, they were unable to read body language and demonstrate empathy through touch or facial expressions.

*With comforting a patient, it’s very challenging to do that on the phone, because what are you supposed to do? So the only thing that you have [are] comforting words… I think it’s very helpful when the patient is there sitting in front of you, especially when you’re breaking that news to the patient as well as the family. Face to face interaction is very important during that time.* (W2G3S2)

A few students also commented on the usefulness of being invisible – hiding behind a phone – particularly when they felt uncertain.

*So in some ways, it’s almost nice to hide behind the phone as it were, in terms of you just choose your words, they can’t see that you’re feeling a bit awkward.* (W1G5S1)

Despite challenges associated with telemedicine, students spoke of how their skills of listening and communicating had improved. This benefit was experienced as they returned to their studies.

*Because it’s just over the phone, I think you’re forced to be a little bit more attentive with what the person is saying. And just pay attention to their tone in order for you to actually figure out their state of mind and to try and help them as best as possible*. (W1G5S2)

*I think my patient communication improved a lot. Because when you’re speaking over the phone, there’s a different kind of barrier compared to talking in person...while still allowing them to feel heard and not cutting them off... And now I’m able to do the same in my in-person clinical activities. And I found that one of the skills I… learned was to try to relay my empathy without being in front of somebody while still asking them quite personal information.* (W2G4S2)

#### Hierarchies and teamwork

Students and healthcare professionals worked side-by-side in multi-professional pod teams. These teams comprised doctors, nurses, social workers, psychologists, physiotherapists, and other health professionals who volunteered their assistance. Besides the pod leaders, there were no hierarchies. Students appreciated the teamwork and the flattening of the usual hierarchy they experienced.

*It was the first time as a medical student that I have ever experienced that kind of flattening of the hierarchy and it was really nice, it made me feel a lot more welcomed and a lot more useful.* (W1G2S1)

*It was really nice to be part of a multidisciplinary team and feel like you’re actually contributing and to be treated like a colleague and you’re basically on the same level as all these people on the group.* (W2G1S1)

The multi-professional nature of the pod reassured students that they were supported and could get assistance or refer the patient to someone else if they were not able to manage issues adequately. They drew on different team-members and learnt from the experience of their peers, as well as the more experienced pod members.

*COVID was a completely new disease that no doctors really knew about. And so it was quite fun to be learning with future colleagues, and you were … all at the same level, where no one knows about this.* (W1G4S5)

*The team worked. You don’t have to suffer alone and you can always ask for help. Even if you don’t know the answer, there’s always someone else who might be able to come up with a solution.* (W1G5S2)

Many voiced that students other than health science students, could be involved with the work. Some suggested that enrolling volunteer students with computational skills could have solved longstanding unresolved technical issues such as wrong phone numbers as well as improved workflow efficiencies.

..*taking psychology, medical students and social work students … they’re the people that are in the field, mostly. And I think we understand better … issues that ha[ve] to do with people’s attitude, people’s characters… Because it would be hard to take someone who doesn’t understand … what support means to someone*. (W1G4S2)

*There are a lot of young students, … particularly engineers, Comm Sci students, people with amazing technology skills, … communication skills … who would probably love to put those skills to use in a pandemic context.* (W1G4S5)

While not a concern expressed more widely, one student remarked that the work ended abruptly and felt incomplete given the intensity of the work and their roles in teams. They suggested instituting a closure event for volunteers.

*We went through quite an intense period where we all worked together, but then we … just stopped… We never heard after that, what happened with the team… So maybe some sort of closure would have been nice.* (W1G5S3)

#### Training and support

Being prepared helped students feel more confident. The online training, videos, and documents, read before starting, oriented them to the scope of work; prepared them to ask specific questions when making calls; and detailed the documentation required, including the reports to the PDoH. Debriefing sessions enabled students to speak about their experiences, their feelings, fears as well as their triumphs. Despite the support and the training, many found their work responsibilities quite daunting.

*I was given some documents of the interview process... I took that document and just typed it out nicely for myself that fit onto one page. So, the first hour of the elective was spent purely on preparing myself… I took that document and I tried to memorize it, because I’m not the most comfortable person with phone calls.* (W2G2S1)

*The debrief session really helped me, because I didn’t want to talk about it, the personal situations on the phone … with my family. But in that situation, a lot of people [were] going through the same thing. So, I felt like I could let those feelings out and it helped hearing a lot of people were either feeling the same way or had gone through similar phone calls and get advice on that.* (W2G4S2)

Suggestions for volunteer training and improved resources, recommended by students who worked over Wave 1, were implemented. These improvements were commented on as strengths by students working over Wave 2.

*We actually were giving more information … than what the media and the general Department of Health has given. One is to make sure that they keep their houses well ventilated during isolation… So have an FAQ for people.* (W1G4S1)

*Training was the old “see one, do one, teach one” training. It wasn’t so formal, and we just … learned on the job. It sounds like they’ve developed it quite a lot, which is great.* (W1G5S3)

Some suggested additional training that would both assure quality of calls and give students more confidence. These included shadowing experienced volunteers and conducting observed simulated telephone calls.

*What would have helped me to be more confident in my contract tracing is some kind of quality control ... Maybe …[a] mock interview would be interesting. Not that I did a bad job… but someone could be doing a bad job … and nobody would know. And I think that’s a problem.* (W2G2S1)

In SA, WhatsApp is the preferred application for communication – between individuals and within groups. Consequently, much of the communication and support in pods was through WhatsApp groups. One group housed essential information, health education material and general communication to be shared with cases. Members of the pod, including students, were able to pose questions on the pod WhatsApp group and obtain an almost immediate response. The group became a powerful source of communication, advice, information, and support. The students felt they could ask any question and not feel “dumb”.

*The WhatsApp group was incredibly beneficial, and I don’t think I would have managed without it. I think it was probably incredibly critical to this whole service functioning and being efficient ... Because then you’re learning from everybody else as well and you’re gaining knowledge from their experience.* (W2G2S2)

The pod acted as a safety-net and offered support beyond the immediate cases students were working on. When students faced a difficult situation or were distressed or going through their own issues, they could contact pod members or leaders for assistance that was readily offered. Students appreciated the kindness shown to them by both the cases they called, as well as pod members. The work was an opportunity to participate in a different kind of patient care, focusing entirely on the circumstances faced by the patient, their family, and their contacts.

*It was interesting that people were so kind in response to me and I loved that space because it felt like for the first time in six years of medicine that it was about the patient fully…I connected with a human being.* (W1G1S2)

Students worked long hours as each call could take up to an hour or more. They spoke of the different ways in which they learnt to cope with the stresses of the work. They got some ideas from pod leaders or team members – listening to music, knitting, reading, walking, to ensure they took breaks between calls.

*It’s very cathartic to have a good rant to someone close to you, the kind of debrief after a call. And … I also agree with making time for hobbies. I love baking and playing music. It’s fun to invest in a hobby that sort of takes your mind off all that’s going on.* (W1G4S5)

### Personal and professional growth

Despite challenges described by students, the opportunity to be part of a C&CT team clearly gave students opportunities to develop and grow personally and professionally.

#### Personal impact

The work had an emotional and physical toll on many students, who typically described it as a “draining” experience as they felt unprepared for the numerous stories of hardship. Students had to hold a supportive space for cases and contacts, but many questioned their own abilities.

*But after a while, it did get quite taxing on me to have people like call me constantly or WhatsApp me and ask me for things. I had one person as well who …messaged me at three o’clock in the morning, and I don’t actually know why I was awake. And I think he wasn’t doing very well… He needed an ambulance… I think I dealt with quite well. And he ended up being fine, but it was … a bit stressful.* (W1G4S3)

*The pressure of providing for these people balanced with how personally uncomfortable and unqualified you feel, is very draining* (W1G1S2)

Despite the challenges, students found making calls and speaking to cases fulfilling. The people called were receptive and spoke openly, often being so grateful to be contacted. This kept student volunteers motivated to make further calls.

*I think a lot of them didn’t expect a phone call. And when you do check in and just ask them how they are doing, what are the symptoms like and give them some information on what they could do to make themselves feel better. Hearing how grateful they were and how much they appreciated that. That motivated me to keep going as well.* (W2G4S2)

*I would never think that complete strangers would be so grateful.... But they were very understanding and, in fact, very thankful that we had… provided support to them as being in food or in the work certificates for their bosses.... they answered everything, and they never really withhold any information when we get asked anything.* (W1G3S3)

Some framed the challenges of the work as an opportunity to learn about the range of illnesses caused by SARS-CoV-2 and how people managed their illness. This was fulfilling as it would assist them professionally.

*Personally, I learned a lot about COVID-19 just working and trying to help the front liners by contacting these cases. You get to learn a lot by experience from one patient to the other because every patient deals with COVID-19 differently. So, I think that was really a good opportunity for me in my career.* (W2G3S2)

The work made many feel professional and responsible as they were a source of knowledge. Ultimately, they had to trust themselves to do a good job.

*Most of the patients that I called, wanted somebody to explain to them what was really going on. Because there’s a lot of myths and misconceptions about COVID-19. So, I got to tell patients the truth about COVID-19 and address the myths, that they heard from friends, or they heard from unreliable sources*. (W2G3S2)

*It was a really great opportunity for us to learn to trust ourselves and to trust the future health professionals that we are becoming.* (W2G1S2)

#### Making a difference

Throughout, students felt positive about having made a real difference in people’s lives. They found it rewarding to alleviate people’s stresses and burdens by linking them to resources.

*Sometimes you feel like you can [help], whether it be speaking through fears or misconceptions, or just reassuring someone or giving them advice on [drugs]. I found [it] quite rewarding, being able to really calm someone’s fears and being able to be that source of information and comfort to someone*. (W1G5S1)

*So being able to … apply for them to go to an isolation and quarantine facility if they can’t do that at home. Or being able to contact someone to send a food parcel or something; or send a field team or something like that. I think that that definitely helped.... at least I can make an impact for that person and hopefully make the experience a bit easier for them.* (W1G3S1)

This work was different to students’ previous interaction with patients during their training, as they perceived their learning role as having little impact. This experience was, in contrast, more meaningful.

*At the end of it, … I was very happy. I genuinely felt like I made a difference. Whereas… sometimes in med school, you’re … just poking and prodding patients and asking them questions and you’re not really doing anything for them, you’re just learning through them.* (W1G3S3)

#### Knowledge and skills

The work of C&CT was a steep learning curve, but also good practice for all students’ future careers. As skills were practiced, students became more confident about their own abilities.

*I feel more confident with interviewing people and made me realize that even if it’s something you don’t feel comfortable with initially, it’s … a skill that improves with practice.* (W1G6S2)

*The skill that I gained was the confidence to be able to just pick up the phone and talk to people … It’s made me more confident in terms of going up to a patient and just speaking to them.* (W2G1S1)

Besides breaking bad news, gathering and communicating essential information, students appreciated the opportunity to learn and practice reflection, problem-solving, organisational, interviewing, and history taking skills to manage patients holistically.

*It’s been an interesting experience. I feel like I’ve gained useful skills in terms of my career. I’d have to call different people, different institutions, try to gain information, people skills in terms of navigating people who might not be having a great day or getting information from people where it’s not their job to help you, but you try and play on the best intention. I don’t know how to say it, but negotiating, basically.* (W2G2S1)

*…grappling with confidentiality issues as well, especially with employees... You have to disclose who the person is because otherwise they won’t be able to trace … who needs to isolate.* (W1G5S3)

Many students thought that making C&CT a formal elective option had worked well for medical students, universities, and the health department. They developed skillsets in telephonic consultations. This was highlighted, as many thought that telemedicine was sure to become a future communication vehicle, for both consultations and health messaging. Some advocated for placements that would allow students to practice core skills such as ‘breaking bad news’.

*the phone calls were a good strategy… I recommend … for future purposes because it’s much of a digital age now.* (W1G2S4)

*But rather [than] just telephonic interviews. Health interviews, I think would be advantageous. You know, high impact, short time and being able to address things through that way.* (W1G3S2)

*Students just have one day in which they do contact tracing so that they can just learn how to break bad news. I think it’s a good learning experience in one’s career. Just have the exposure.* (W2G3S2)

#### Privilege

Students expressed concern about the living circumstances of the people they called. Abject poverty was evident as many cases and contacts lived in desperate circumstances of overcrowded homes and limited access to sanitation facilities, and taking time off from work would mean no income for themselves or their families. This was in stark contrast to most students’ own life experience and highlighted the vast differences in socio-economic circumstances across the city and country. They reflected on their own privilege and discomfort when asking personal questions of cases, whose socio-economic challenges contrasted to their own life experiences.

*What was also really challenging is hearing people’s living conditions. And then trying to advise someone to isolate back where they live with multiple other people? You’d call someone and .. “Well, you can’t go to work for … two weeks”. And they’d be, “I have to go to work, otherwise, I don’t have food. I don’t have money for food”… So that was really tough… and challenging too, to actually hear about...* (W1G3S1)

*Getting to know people’s living situations was not easy. I think a particular experience for me was … it was a household of ten. And … two bedroom and one bathroom, but the case actually refused to go into isolation. So, he literally just stayed in one room and decided to use a bucket and a face cloth to shower.* (W1G3S3)

Students gained insight into communities and individuals’ lives, as making calls to people with COVID-19 exposed them to the social determinants of health.

*It was just really interesting… [to] get a look into other people’s issues and the challenges that they were facing. And then of course, to help them even if just in a small way.* (W1G2S1)

*Just having that those calls, they were kind of like a window to the outside of what’s actually happening and in other people’s life... For example, hearing about ten people living in one flat ... it’s hectic out there.* (W1G6S1)

Students often felt humbled by their experience and were left with an awareness of their own privilege in society.

*I think it’s made me very aware of … my privilege and how much I have to be grateful for. Because a lot of the time when we were phoning people about the difficult circumstances about how many people are staying in their house, or how they might not have any support from family.* (W2G4S2)

Some remarked how people’s living conditions impacted on viral transmission. They maintained that adding health resources – personnel and systems – together with changes to social conditions that facilitated viral transmission, such as overcrowded housing, could have limited the pandemic locally. COVID-19 exposed social inequities.

*If problems such as housing could be like taken care of, there will be less overcrowding, therefore, the spread would be less... If there are more health care workers*. (W2G4S3)

#### Authority

The back-up provided by the pod gave students the confidence to be able to speak with authority. They were asked to introduce themselves as being from the Department of Health, which gave them confidence and many felt like qualified health professionals.

*It is quite strange speaking to people a lot older than you and increasingly having this authority of knowledge of being a health professional.* (W1G1S4)

While having the authority of the PDoH behind them, students felt an enormous sense of responsibility to give of their best, not only to the patients but also to their peers on the pod.

..*the responsibility of knowing that you have to get it done. And so the next team can do their job, you have to be diligent about doing your own…typing out everything, making sure everything makes sense. Everything is submitted on time. So, there was a lot of fulfilment in doing that.* (W1G2S2)

A few expressed concerns about their role as part of the team, representing the PDoH when they contacted cases. They highlighted that those called assumed that they were doctors. This left some feeling like imposters which reinforced their sense of being out of their depth.

*People just assume you are a doctor* (W1G1S5)

*Every time I introduced myself as [] from the Department of Health, … is this really the way I am supposed to introduce myself? I felt a bit of a fraud when I did that* (W1G1S1)

*I definitely felt like an imposter in the beginning, because I just went from being like a medical student and now suddenly on the phone with people... a lot of pressure to make sure you’re providing the right info and as much as you can learn beforehand... we were one of the first batches when they called for volunteers from the younger classes. So yeah, I just felt very much like a bit out of my depth and… I hoped I could provide this necessary skill.* (W1G6S2)

Some raised that over time their involvement in the work led to a certain status. They became a resource in their own families and communities where they were able to share knowledge and information.

*I talked to some of my family members who had COVID-19. And other people in my life were like, asking questions about COVID-19, because I already did contact tracing. So, I knew how to advise them and it… made me more of an advocate in terms of assisting people and giving them information.* (W2G3S1)

#### Reflective moments

While many students benefited in their development as future health care professionals, the work also led to personal growth, providing all with opportunities to reflect on the kind of work they desired as future professionals. Additionally, long conversations with seriously ill cases made them think about managing patients’ mortality, their own lives and spirituality.

*There was this one woman with comorbidities I was talking to. At the time, she was feeling well, but we … dialled into her conversation about her mortality, and her fears, surrounding … her other comorbidities and how they may affect her illness… So, it was also emotional, … talking to someone with the real … fear of dying. And… you can never really say that “you’re not going to” or that “everything is going to be fine”. But you also don’t want to scare them, you still want to reassure them. So I think it … shaped my understanding of … the limited time that we have on this earth, and in a way just made me think more about the spiritual aspects.* (W2G4S1)

#### Public health in action

Some students highlighted the benefits of learning about public health and health systems. These are often not valued by health sciences students who are largely orientated towards direct clinical work with individuals. Exposure to public health is often theoretical or research orientated, and students do not have much service exposure to working practically in public health orientated projects. They believed that the C&CT exposure was important as it offered students an opportunity to see ‘public health in action’ – both the exposure to public health functions and understanding the epidemiological trajectory of the pandemic. They recommended that more practical public health training should be included in curricula and remarked how they appreciated the opportunity of being involved.

*It was really lovely to be part of a public health response...it was really great in terms of getting that practical exposure.* (W1G3S1)

*Include, even if there isn’t a pandemic, some sort of public health practical training... as part of our actual core curriculum so that we can get a good understanding of it, because it is really important*. (W2G4S1)

A few students also reflected that the experience had given them a different perspective and greater appreciation of the work of the Department of Health.

*…my perspective of the Department of Health. I feel like they all care more than I have been led to believe as a med student. As a general member of society, you learn a lot of negative stories… And having actually a positive experience where you feel like you are working for a government that cares.* (W2G2S1)

## Discussion

While the COVID-19 pandemic challenged health systems globally, it provided an opportunity to re-imagine health services and health sciences education to find novel ways to respond to health priorities. As was experienced in better resourced health systems, the COVID-19 case investigation and contact tracing strategy, while being core to the provincial response, put a huge strain on existing human resource capacity. This resulted in calls for volunteers, and consequent deepening partnerships with universities. As was highlighted in the USA [3, 24–27], student volunteers became a valuable resource and formed the basis of volunteer teams working across Cape Town. Pre-existing partnerships with local universities enabled students to work under supervision. Student volunteers, were eager to participate in C&CT [28], and formed the basis of multi-professional volunteer teams, benefitting the services.

In view of the involvement of student volunteers in this initiative, this study explored students’ motivations for volunteering, their experiences of C&CT and recommendations with the purpose of making policy and educational recommendations. Three main themes were generated from analysing the FGDs – students’ motivations for involvement, their experiences of navigating roles and responsibilities, and their personal and professional growth.

Students needed to adapt to autonomous on-line learning which is vulnerable to insufficient guidance and feedback. These are required to achieve competence, together with peer communication to ensure relatedness [29].

### Motivations

Students were motivated by the opportunity to work from home, but more importantly to make a difference and improve their skills. They wanted to help to combat the epidemic and to support and positively impact on cases and their families’ lives. Similar to the experience in Nebraska [26] and Yale [3], students wanted to contribute to impacting on the trajectory of the pandemic, while gaining experience and professional exposure. In this study, students were driven by their identity as health-professionals-in training, and from seeing COVID-19’s impact on family and friends. This experience was also reported in a New York (NYC) study among contact tracers [30], who felt honoured to help others from the comfort of their home. Working from home allowed students to work autonomously and make time for self-care, a factor also valued by public health workers reported in a USA study [31]. Interestingly, unlike in the cross-sectional study in Indonesia, students in this study were not afraid for their own health, but prioritised protecting the health of their family members [32].

Students’ motivations for volunteering for C&CT were realised through their experiences. They had to assume new roles and take on responsibilities outside the observer or apprenticeship model of learning they had largely been exposed to. They were able to use their clinical knowledge and, like the Nebraskan public health students [26], performed physician roles including identifying risk factors, such as diabetes, for severe COVID-19 and educating such cases to be vigilant and act early for signs of deterioration.

### Navigating roles and responsibilities

Students had to work using phones, largely autonomously, to deal with anxious, socially insecure cases and their contacts and manage work-personal boundaries. Additional challenges arose from their roles and responsibilities including solving logistical challenges, including finding correct numbers for cases, getting food parcels delivered on time, dealing with difficult employers and overcoming language barriers. Despite these difficulties, students felt protected from the potential negative mental health impacts through the support and camaraderie offered [33], and their sense of relatedness [29]. They used social media – the WhatsApp groups – to enhance their learning, for mutual support and dissemination of information about COVID-19, a useful learning modality highlighted in the recent Education literature [34]. They were energised and supported by their experience of teamwork, the absence of hierarchies, and through training and debriefing.

As was the experience in Arizona [35], with the requisite training, students were able to manage the work arising from the case engagement that holistically addressed the person’s needs. Uniquely in this study, over and above direct case engagement, students provided letters for work/school, dealt with employers, ordered food parcels for the food insecure, arranged isolation facilities for those both needing and agreeing to these and, requested home visits if cases were uncontactable or there were concerns about adherence to isolation. The need for such resources was also highlighted by contact tracers in NYC [30], and is noted to be core to the value of engaging in C&CT in disadvantaged communities in the USA and Europe [36, 37].

It is important for contact tracers to communicate in people’s preferred languages [38], and communication challenges were highlighted by some. Yet without available medical interpreters, training and a toolkit, as is advocated in the USA [38], students in this study showed initiative and adopted alternatives such as enlisting cases’ family members to interpret or asked for assistance from other pod members who were fluent.

Students were often confronted by the difficulties of overlapping roles with other health professionals, changes to COVID-19 isolation policies as well as cases who could not be contacted by telephone. These complications were also highlighted in USA studies [30, 31]. Students overcame these challenges in part due to the teamwork they experienced in the pods, together with the debriefing and the ongoing training sessions that were incorporated into the work programme. As found elsewhere, [34] they valued the online workgroups that used social media for communication and provided immediate response to dilemmas faced or questions posed. A 2020 national USA study of public health personnel working on COVID-19 found that teamwork, cooperation, camaraderie and managerial support protected their mental health in a context of high work demands, and the unchartered territory of COVID-19 control. As found in this study, they reported that opportunities to “decompress with colleagues having the same experiences” were valuable [31]. Astute supervision was offered, a requirement for learning that develops competence [29]. Supportive supervision that is available and responsive was also noted as an enabler for C&CT personnel in the USA [30].

The importance of training elements such as clear instructions, scripts and reporting instructions together with confidentiality agreements is noted in USA C&CT programmes [37] and in Indonesia [32]. Students proved to be client-centred, attentive listeners, providers of holistic care, who educated and advised individuals and communities. Such roles for higher education institutions were highlighted in the pandemic’s first year [39], and accords with being a socially responsive educational institution [40]. Students demonstrated empathy, compassion, confidentiality and the ability to build trust. These are emphasised as core communication skills for contact tracers to quickly interrupt viral transmission [9, 41], and were noted as the C&CT training focus for people with no prior health work experience [9]. This vital communication role is echoed in research from the USA, that noted that contact tracing work was a “mix of first responder health educator/advisor and social worker”[30]. Social Workers advocate for the inclusion of motivational interviewing into other professional training [42], as these skills are well suited to C&CT tracing work. This was implemented in the USA and these skills are taught to medical students at the University of Cape Town. Students recognised their prior skill sets, acquired during their health professional studies, that prepared them for C&CT. They adapted well to new learning methods, using their new-found knowledge, honing skills, demonstrating values (such as being social responsive), and attitudes such as compassion. This signals that both deep learning and key elements of competency based education [43] were achieved.

### Personal and professional growth

Students felt fulfilled by the knowledge that they were able to make a difference and gain valuable skills, even though, as described in a NYC study [30], the work had a physical and emotional toll on them. They were acutely aware of their positions of privilege and authority but used this to reflect on the disadvantage they witnessed and learnt from seeing public health in action.

Similar to the report from Washington [24], C&CT provided an opportunity for experiential learning where all students could both consolidate clinical skills and develop new skills in telephonic consultations and public health. The opportunity for student learning provided by COVID-19 C&CT has been advocated by university teachers in diverse settings [26, 35]. Students in this study saw the value of the experience for professional growth, improving their clinical skills, which was also noted by a medical student in the Nebraskan programme [26]. Students advocated for including such opportunities into curricular activities. In the Connecticut study [3], researchers suggested curricular changes such as including C&CT in student practicum options.

These findings contrast with findings from a quantitative study investigating learning impacts of COVID-19 among students in four diverse country settings [44]. While those students were able to adapt to online or hybrid learning and were self-efficacious, many – particularly Omani students – felt unengaged and unsupported and their sense of well-being suffered. In this study, students found meaning and learnt with and through a connected, experienced multi-professional team. This highlights that attention must be given to communication modalities with supportive supervision, to optimise learning.

Students in this study demonstrated insight that a range of skilled personnel are required to manage a societal health crisis such as COVID-19. They recommended that other student volunteers, with technical skills, could have been recruited to assist with developing data systems and analysis, which were needed. Students with these skills were recruited for such work in Nebraska [26].

Several studies have recognised the added difficulties that poverty-stricken communities faced because of the COVID-19 pandemic [2, 9, 30, 36, 45]. The demands of isolation exacerbated social inequities. Mooney, a health historian, noted that the wealthy are privileged to quarantine at home [41]. He advocated that the cost of compliance with public health measures should be addressed through access to welfare, food banks and other support, which were offered, albeit inadequately, in this C&CT programme. Some students were economically disadvantaged and struggled with the cost of internet connectivity and airtime, essential for the work. Consequently, arrangements were made for them to use campus WiFi, have their airtime reimbursed by government and later, used VoIP telephony. Support for vulnerable students is key to the success of online or hybrid learning [39], and universities must give attention to reducing “digital divides, promoting sustainable activities” [46].

### Implications for health services and student training

This study demonstrates that, with support, volunteers, such as health science students, can be drawn into critical health sector work to supplement inadequate numbers of skilled personnel, providing holistic care. The value and importance of mobilising such resources in emergencies was also highlighted by a multi-country African study [47].

The experiences of the students in this study show the value of the C&CT programme for helping students to apply their clinical knowledge together with learning new public health skills, to develop competence. It points to an opportunity for health sciences faculties to re-examine curricula with a view to enriching clinical and public health learning not offered in current curricula. For example, as self-reflection on one’s own privilege and appreciation of patients’ belief systems and spirituality underlie provision of holistic care of gravely ill patients, these should be incorporated into curricula. Additionally, as telemedicine became a vital vehicle in patient consultations during the pandemic [48], incorporating telemedicine skills and services delivered by students under supervision into curricula can equip future professionals for the practice context they will encounter. As suggested by students, involvement in health messaging programmes, such as telephonically following up patients who are lost to follow-up for chronic diseases including HIV, TB or diabetes care, key conditions in SA and elsewhere, would enable future students to gain insights into public health functions, social determinants of health, health promotion and prevention, and prepare them for practice.

Findings highlight elements critical for the success of future online or hybrid courses and attention must be paid to student autonomy, competence and relatedness [29]. Learning was perceived as relevant for the work and for future practice – students were orientated, given access to training material, mentored and supervised; and, communication and support was facilitated through acceptable social media, and facilitated debriefing sessions. These components must be factored into remote, telephonic, health service orientated curricula and practice.

Globally, public health is not typically visible to the public, in health systems and to health sciences students, but COVID-19 highlighted public health functions and its value [33]. Students embraced C&CT as ‘public health in action’ and advocated for its inclusion in medical curricula. Additionally, students spoke of their roles in families and communities through C&CT and felt they were accessible with cases seeing them as trustworthy and knowledgeable, factors critical for effective patient care. They were important vehicles for providing public health messaging as was reported in other studies [35]. The value of health authorities utilising accessible, trustworthy channels for health information was also highlighted in research on COVID-19 from Michigan [49].

Researchers in other studies conclude that students who were part of C&CT volunteer programme experience gained skills that should make them more rounded [25] and more effective [35] when they enter the workforce as health professionals. The skills set which students in this study developed included interviewing, problem-solving and organisational skills coupled with cultural competence, reflectivity and systems thinking. These should prepare them for becoming confident leaders, able to navigate challenges they will encounter as young health professionals. These proposals pre-suppose trusting partnerships between health departments and universities attuned to the requirements for remote learning.

Changes proposed for curricula include providing opportunities for all health science students to gain skills in telephonic consultations. However, remote telehealth learning for health sciences students, needs carefully planning, particularly in LMIC settings. Implementation may require the provision of additional resources such as appropriate devices and connectivity for students; and committed and experienced staff open to interdisciplinary collaboration and developing new skills themselves. Such learning could be integrated into existing courses. Staff would orientate students, formulate clear learning objectives, devise appropriate assessment methods; offer attentive support, and promote students’ connection through, for example, social media. Such initiatives would need to be researched and evaluated. Then, health science students would not only become excellent clinicians, but also appreciate and participate in public health work that benefits communities, learning outcomes valued globally.

### Limitations

This study did not intend to describe the output or impact the students working in C&CT made on the health system, through analysing caseloads or cases traced. It is therefore not possible to report on the numbers of cases called or contacted, nor can the extent of wrong numbers furnished for C&CT purposes be verified. This was a qualitative study that intended to explore the experiences of students with a view to making recommendations for the health system and student learning. We did not explore the costs of such a programme and recognise that some students may have been unable to participate in the initiative due to economic constraints. Our participants may not represent the experiences of other students who while participating in the C&CT programme, did not respond to the invitation to participate in the research. Nonetheless, reports on student involvement and experiences on university websites [10, 50] accord with study findings. In addition, the experiences of those interviewed were largely congruent signifying transferability – that the experiences reflected at least a large section of WC student volunteers.

## Conclusions

This is the first qualitative study on health sciences students’ experiences of participating in a C&CT initiative over the COVID-19 pandemic from Africa, and their learning. While C&CT was not able to interrupt SARS-CoV-2 transmission in SA, it may have provided support to cases as evidenced by students’ experience of the gratitude expressed by those contacted.

The limits to contact tracing and roles are discussed in the literature and this varies from its effectiveness “at the start of a pandemic or supressing outbreaks” to once case numbers have decreased [2]. While there are limits to C&CT for COVID-19, it has value if social determinants of health are explicitly addressed – such as employment and food insecurity – as well as using the opportunity for empathetic listening which mitigated the mental health distress associated with a COVID-19 diagnosis [51]. Indeed, students’ work in C&CT focused on building relationships in addition to surveillance.

Students were and can be integral to C&CT systems that require community engagement, planning, consideration of local contexts, training, technology such as telephony, and systems to compile and analyse data in real time [37]. This was an opportunity that benefited students, the health system, and universities. It was a well-designed and powerful learning opportunity that factored in student autonomy, relatedness together with competence [29]. This enabled students’ experience of agency and developed their clinical, public health and personal skills. The health system gained personnel and piloted initiatives that drew on future health professionals, which demonstrates that that health services can gain from trusted academic partners.

Students’ rich experience provided opportunities for reflection and growth. As was central to this initiative, there are opportunities for educational institutions globally, to intentionally provide exposures for student learning that span professional boundaries, which simultaneously expose students to individual patients and public health functions. These would enable health sciences students to be service ready, contributing to communities’ health.

## Electronic supplementary material

Below is the link to the electronic supplementary material.

Supplementary Material 1

## Data Availability

The datasets generated by this qualitative study and analysed during the current study are not publicly available due study participants being assured of their confidentiality and anonymity. However, they may be available from the corresponding author on reasonable request.

## Abbreviations

COVID-19: Corona virus disease due to
C&CT: Case and contact tracing
FGD: Focus Group Discussion
PDoH: Provincial Department of Health
SA: South Africa
SARS-CoV-2: Severe acute respiratory syndrome coronavirus 2
USA: United States of America
