# Supplementary material for: Health Sciences students’ experience of COVID-19 case management and contact tracing in Cape Town, South Africa
Source: BMC Med Educ. 2023 Apr 12;23:238. doi: 10.1186/s12909-023-04205-4 (PMC10092931; doi:10.1186/s12909-023-04205-4)
Supplement: Supplementary file 1 — Supplementary Material 1 [file 12909_2023_4205_MOESM1_ESM.docx]

**FOCUS GROUP GUIDE**

**Welcome and introductions**

Welcome everyone to our focus group in which you will be invited to reflect on your experiences, thoughts, feelings and recommendations related to case and contact tracing.

My name is …. I shall be facilitating today’s focus group, supported by ….. We had the pleasure of working with many of you in the various Pods and we are looking forward to hearing about your experiences.

Have you all received, signed and returned your consent form? *(If no, take participant through the main issues and send it to the participant immediately after the focus group)*

**Contract**

Before we begin, I would like to clarify a few things:

- Purpose – focus on three areas – how you became involved, your experiences of case and contact tracing, and recommendations you have based on your experiences
- Roles of interviewers and participants – specify how we will facilitate the discussion and takes suggestions from the group. Clarify expectations.
- Ground rules – discus how best to facilitate the session including input from participants.
- Confidentiality – explain recording. Transcription will be anonymous. Group to contract confidentiality.
- Duration of focus group – maximum of 90 minutes including time at the end for any questions or concerns

**Questions**

Let’s begin….

Looking back on your experiences of case and contact tracing,….

1. **Please tell us how and why you got involved in COVID-19 case and contact tracing.**

What prompted you to take on this COVID-19 role?

1. **Let’s move onto discussing your experiences of case and contact tracing. What stands out most for you as you think back on your experiences?**
2. Share with us your thoughts and feelings about COVID-19 and case and contacting.
3. Tell us about your preparation for your COVID-19 work.
4. How did you feel then, and now, about your COVID-19 work?
5. Is there anything that enabled you to do your COVID-19 work?
6. Tell me about any differences between your COVID-19 work and your clinical rotations up to now.
7. Did you experience any challenges concerns in your COVID-19 work? And how did you respond to these?
8. Is there anything you needed to be effective in your COVID-19 work?
9. Has anything changed in your life because of your COVID-19 work?
10. Share with us your experience of receiving support (internal and external) for your COVID-19 work?
11. Tell us about your insights and concerns in the face of the pandemic.
12. Share with us your views of the available information for the public about COVID-19 care and control measures.
13. **Based on your experiences, what sorts of recommendations do you have for future case and contact training**
    1. Is there anything that Government should stop doing to strengthen COVID-19 care and control measures?
    2. Do you have any recommendations for enabling collective COVID-19 care and control measures?
    3. Do you have any recommendations to the university about these experiences for students in the course of their training?
14. **Have we missed anything? Is there anything you would like to add before we end our discussion?**
15. **Thank you so much for giving of your time.**

***Probing questions will be used to obtain clarity and enhance the depth of discussion***

*These will be open-ended questions starting with “who”, “what”, “why”, “when”, “where” or “how”, and “please tell me more about that”*
